# Mechanism and management of atrial fibrillation in the patients with obstructive sleep apnea

**DOI:** 10.1002/joa3.12784

**Published:** 2022-09-27

**Authors:** Yu‐ki Iwasaki

**Affiliations:** ^1^ Department of Cardiovascular Medicine Nippon Medical School Tokyo Japan

## Abstract

Obstructive sleep apnea (OSA) is a highly prevalent disorder in patients with atrial fibrillation (AF). Although there has been an increase in the incidence of AF due to the aging population, it has been reported that OSA is still underdiagnosed because many patients remain asymptomatic or unaware of the symptoms associated with OSA, such as daytime sleepiness. Untreated OSA reduces the effectiveness of AF treatment, regardless of pharmacological or non‐pharmacological modes of therapy, such as catheter ablation. Experimental and clinical studies have shown that OSA pathophysiology is multifactorial, comprising of hypoxemia, hypercapnia, autonomic dysfunction, negative intrathoracic pressure changes, and arousals of OSA, and lead to AF. Both the acute and long‐term effects of obstructive apnea episodes are involved in the development of an arrhythmogenic substrate of AF. Undiagnosed OSA causes underutilized opportunities for more effective AF management. Therefore, it is important to screen for OSA in all patients being considered for rhythm control therapy. However, regardless of the growing evidence of the negative prognostic impact of OSA, there is a lack of awareness regarding this connection not only among patients but also among cardiologists and arrhythmia specialists. There is a barrier to performing a systemic screening for OSA in clinical practice. Therefore, it is important to establish a comprehensive OSA care team for the efficient diagnosis and treatment of OSA. This review provides the current understanding of OSA and its relationship to AF and the importance of the diagnosis and management of OSA in AF.

## 1 INTRODUCTION

The prevalence of atrial fibrillation (AF) has increased with the aging population in Japan.^1^ Hypertension, diabetes mellitus, heart failure, coronary artery disease, and chronic kidney disease are reported to be independent risk factors for the development of AF.^2^, ^3^ In a previous study, the prevalence of AF in patients with obstructive sleep apnea (OSA) was 4.8%, which was higher than that in the control group (1.9%).^4^ The increased incidence of OSA was also related to the increase in new‐onset AF. In contrast, the prevalence of OSA in patients with AF is reported to be 50–80%.^5^, ^6^, ^7^ Notably, a cohort of patients who underwent catheter ablation for AF showed a high prevalence of sleep apnea.^8^ Coexisting OSA reduced the maintenance rate of sinus rhythm after catheter ablation and electrical cardioversion in patients with AF.^9^, ^10^, ^11^ In clinical practice, it is essential to identify the presence of concomitant OSA for the effective management of AF, in order to prevent its recurrence and associated comorbidities. Moreover, the number of catheter ablations performed to treat AF has been increasing over the last two decades^12^, ^13^ because of the advancement of 3D mapping systems and ablation catheter technology, including balloon ablation. International guidelines recommend catheter ablation for achieving rhythm control in the management of drug‐refractory symptomatic AF.^2^, ^13^, ^14^ Appropriate periprocedural management with a correct understanding of OSA is important to determine the long‐term efficacy and safety of catheter ablation for AF. The present study reviews the current understanding of OSA and its relationship to AF and the importance of diagnosis and management of OSA as a part of the comprehensive treatment for patients with AF.

## 2 PATHOPHYSIOLOGY OF AF IN OSA

**FIGURE 1:**
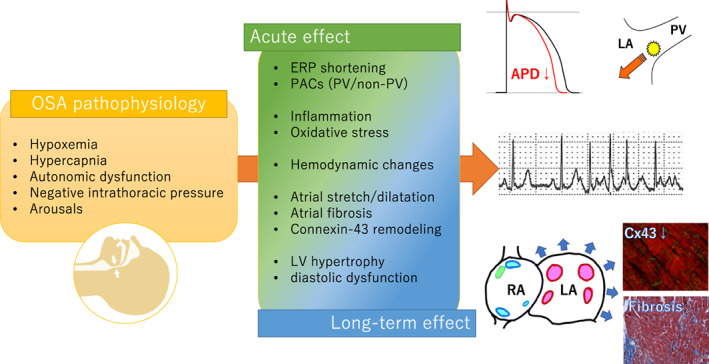
Suggested mechanisms linking OSA pathophysiology and arrhythmogenic substrate of AF. Multiple and complex OSA pathophysiologies develop both acute and long‐term arrhythmogenic substrates, leading to AF trigger and perpetuation. APD, action potential duration; Cx43, connexin‐43; ERP, effective refractory period; LA, left atrium; LV, left ventricle; OSA, obstructive sleep apnea; PV, pulmonary vein; RA, right atrium.

OSA causes hypoxemia, hypercapnia, autonomic dysfunction, arousal, and substantial negative intrathoracic pressure changes (Figure 1). The pathophysiology involved in OSA elicits inflammation, endothelial dysfunction, coagulation imbalance, hemodynamic alterations, electrical/structural remodeling of the atria/ventricle, and autonomic dysregulation. These factors are associated with AF initiation and perpetuation. Therefore, the pathophysiology of OSA is multifactorial and many unresolved complex mechanisms are involved in the development of AF, thereby causing both acute and long‐term effects on its arrhythmogenic substrates.

The acute effects of OSA are mainly attributed to electrical remodeling (electrophysiological changes), and the long‐term effects of OSA cause both electrical and structural remodeling. Several possible mechanisms associated with the electrical and structural remodeling leading to the occurrence of AF have been reported.

During upper airway obstruction, negative intrathoracic pressure becomes prominent, reaching pressures of less than −50 mm Hg.^15^ Esophageal pressure during catheter ablation of AF with deep conscious sedation was −40 mm Hg without airway management.^16^ Fluctuation of the negative intrathoracic pressure causes electrophysiological alterations through various mechanisms. Rostral fluid shift in the supine position also exacerbates upper airway obstruction.^17^, ^18^ Deeply negative intrathoracic pressure increases venous return, impairs left ventricular (LV) filling, and diminishes stroke volume. The redistribution of the circulating blood volume to the intrathoracic cavity causes dilatation of the atria. Acute atrial stretch causes shortening of the effective refractory period along with slowing of the conduction velocity across the pulmonary vein (PV)–left atrium (LA) junction.^19^ Moreover, strongly negative intrathoracic pressure activates intrathoracic baroreceptors, inducing autonomic reflex responses and causing refractory period shortening that also promotes AF. An animal model of sleep apnea showed effective refractory period shortening with increased AF inducibility during obstructive apnea.^20^, ^21^ In an obstructive apnea canine model, AF was induced reproducibly by turning off the respirator during end expiration for 2 min. The AF inducibility could be eliminated by ganglionated plexi ablation or pharmacological autonomic blockade.^22^ Negative tracheal pressure in a pig model increased AF inducibility and shortened the atrial refractory period, which were prevented by the administration of atropine or vagotomy.^21^ Atrial tachyarrhythmia could be induced by atrial burst pacing in obstructive apnea model using Zucker obese rat. Pretreatment with atropine and propranolol prevented AF induction in approximately 50% of rats.^20^ Therefore, autonomic nervous activity, at least in part, plays a role in AF inducibility in the acute phase of OSA. While acute hypoxia might contribute to AF progression, hypoxia alone failed to induce AF in the rat model. An experimental study using rats with obstructive apnea showed repetitive premature atrial contractions during obstructive apnea with deep negative esophageal pressure.^23^ It has been reported that AF is initiated during or just after the obstructive apnea episode,^24^ implying acute episodes of OSA acutely enhance the triggering of premature atrial contractions, which can initiate AF.

**FIGURE 2:**
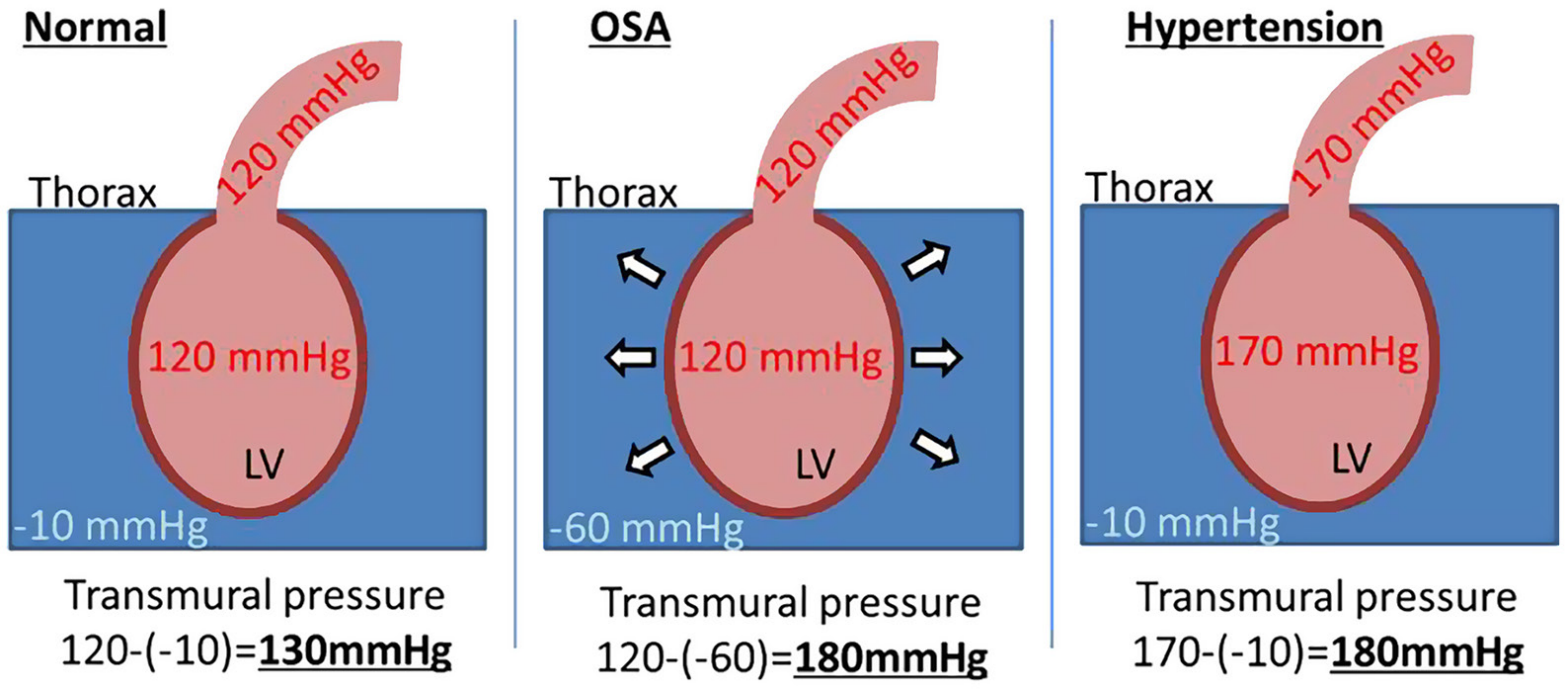
Association between intrathoracic pressure and LV transmural pressure. Patients with OSA presenting with a deep negative intrathoracic pressure of −50 mm Hg and normotension (systolic blood pressure = 120 mm Hg) had a ventricular transmural pressure of 170 mm Hg. This condition is similar to that in patients with hypertension (systolic pressure = 120 mm Hg) without normal negative intrathoracic pressure (−10 mm Hg). OSA: obstructive sleep apnea, LV, left ventricle.

Structural remodeling due to the long‐term effects of OSA plays a crucial role in AF promotion. Substantial negative intrathoracic pressure also contributes to structural remodelling. Transmural pressure is one of the suggested mechanisms of cardiac remodelling in patients with OSA. Even in patients with normal systolic blood pressure (120 mm Hg), OSA with deep negative intrathoracic pressure of −60 mm Hg increased the transmural pressure to 180 mm Hg during the night, which is similar to the etiology in hypertensive patients (Figure 2). Sustained repetitive episodes of OSA may elicit not only atrial remodeling but also ventricular remodeling (LV diastolic dysfunction and hypertrophy) through increased transmural pressure.^25^, ^26^ In addition, hypoxia‐hypercapnia induces sympathetic activation, systemic inflammation, and oxidative stress, which cause LV remodeling.^26^

Long‐term OSA causes atrial fibrosis and reduced connexin‐43 protein expression with the redistribution of lateral cell margins. Optical mapping image analysis showed a reduction in the conduction velocity of the atrium in a long‐term OSA rat model.^27^ Indeed, a clinical study revealed an advanced LA low‐voltage zone with extra‐PV foci initiating AF in patients with AF and OSA.^28^ A recent experimental study with a chronic rat OSA model showed transiently increased atrial exudative stress, which could recover within 24 h. However, 3 weeks of intermittent negative upper airway pressure induced AF promotion by transesophageal atrial burst pacing and caused structural remodeling, including atrial fibrosis, cardiomyocyte hypertrophy, interstitial expansion, and reduced connexin‐43 expressions. These observations were confirmed even in mild‐to‐moderate OSA with high night‐to‐night variability.^29^ Animal studies have shown that chronic intermittent hypoxia increases atrial fibrosis, shortens the refractory period, and increases AF inducibility.^30^ Hypercapnia also contributes to AF vulnerability via slowing of the conduction velocity.^31^ Clinical studies have shown that nocturnal hypoxemia and pulse rate variability are independent predictors of AF.^32^ The progressive nature of atrial structural remodeling along with electrophysiological remodeling associated with repetitive OSA episodes every night promotes AF in the absence of appropriate therapy.

## 3 EVALUATION AND DIAGNOSIS OF OSA SYNDROME IN THE PATIENTS WITH AF

Symptoms of OSA include snoring, daytime sleepiness, general fatigue, nocturia, and morning headaches. There are several questionnaires, including the Berlin questionnaire,^33^ STOP‐Bang questionnaire,^34^ STOP questionnaire^35^ and the Epworth sleepiness scale,^36^ for detecting OSA. A meta‐analysis revealed that the STOP‐Bang questionnaire had superior diagnostic accuracy for OSA.^37^ The STOP‐Bang questionnaire, consisting of eight questions, is often used to screen for OSA (Table 1). The sensitivity and specificity of STOP‐Bang for the diagnosis of moderate‐to‐severe OSA were 92.9% and 43.0%, respectively.^34^ A body mass index (BMI) cutoff value of >35 kg/m^2^ can be used in Asian populations, and lower BMI cutoffs did not improve diagnostic accuracy. The STOP‐Bang questionnaire can be used for multiethnic Asian populations.^38^ However, the low specificity of the questionnaire results in a higher number of false‐positive cases, leading to unnecessary polysomnography (PSG) testing. Approximately 50% of patients with OSA are asymptomatic, leading to underdiagnosis.^39^ These patients are unaware of the presence of OSA. Notably, despite the high prevalence of OSA in patients with AF, most patients do not show daytime sleepiness or unrestful sleep.^40^, ^41^ Thus, self‐reported questionnaires on OSA‐related symptoms seemed insufficient for detecting OSA in patients with AF.^42^ It is recommended that the questionnaire not be used for the diagnosis of OSA but rather to increase the pre‐test probability of suggesting OSA in clinical practice. As can be seen by the question “Has anyone observed you stop breathing or choking/gasping during your sleep?” in STOP‐Bang, it is useful to ask the patient's family or bed partner such questions. By referring to the questionnaire, physicians should suspect OSA while managing patients with AF.

**TABLE 1:** Mechanisms of atrial fibrillation in the patient with obstructive sleep apnea

Snoring?	Do you snore loudly (loud enough to be heard through closed doors or your bed‐partner elbows you for snoring at night)?
Tired?	Do you often feel tired, fatigued, or sleepy during the daytime (such as falling asleep during driving or talking to someone)?
Observed?	Has anyone observed you stop breathing or choking/gasping during your sleep?
Pressure?	Do you have or are being treated for high blood pressure?
BMI?	BMI more than 35 kg/m^2^?
Age?	Age older than 50 years?
Neck size Large?	Is your shirt collar 16 inches/40 cm or larger (measured around the Adam's apple)?
Gender?	Are you a man?
	OSA—Low Risk: Yes to 0–2 questions, OSA ‐ Intermediate Risk: Yes to 3–4 questions OSA ‐ High Risk: Yes to 5–8 questions or Yes to 2 or more of 4 STOP questions + male gender or Yes to 2 or more of 4 STOP questions + BMI > 35 kg/m^2^ or Yes to 2 or more of 4 STOP questions + neck circumference 16 inches/40 cm

Source: Used with permission from http://www.stopbang.ca/osa/screening.php ^60^

The International Classification of Sleep Disorders 3rd edition (ICSD‐3) defines OSA as follows (Table 2). According to the ICSD‐3 criteria, patients who have symptoms such as sleepiness or fatigue or those with comorbidities such as hypertension or AF even without symptoms and five or more predominantly obstructive respiratory events per hour, as observed by PSG or out‐of‐center sleep testing (OCST), can be diagnosed with OSA. PSG is the gold standard for diagnosis, and OCST is considered an equivalent diagnostic tool comparable to PSG. Although OCST is an obstructive apnea event evaluated per hour of the total monitoring period, the PSG is evaluated during sleep. Therefore, OCST might underestimate apnea events due to a lack of total sleep time data and respiratory effort‐related arousal events. Patient education regarding the handling and equipment of sleep testing devices is also important for accurate data acquisition. Despite these limitations of OCST, there were no significant differences in the functional outcome and effect of continuous positive airway pressure (CPAP) therapy and adherence between PSG and OCST.^43^, ^44^ The use of OCST instead of PSG for the diagnosis of OSA might have potential benefits in terms of cost and simplicity in routine clinical practice. Although the OCST has been widespread in clinical practice in Japan, the indication for CPAP therapy covered by insurance should be an apnea hypopnea index of 40 or more in symptomatic patients. Currently, the OCST can be used for the screening of OSA in patients with AF regardless of symptoms. Additionally, a full PSG test is required to confirm the indications for CPAP. Because of the night‐to‐night variation in apnea episodes, repeated measurements may be useful for the diagnosis of OSA. We should always remember that “Negative proof is not proof of negative”.

**TABLE 2:** Left ventricle transmural pressure in obstructive sleep apnea and hypertension

A and B or C satisfy the criteria
A. The presence of one or more of the following The patient complains of sleepiness, nonrestorative sleep, fatigue, or insomnia. The patient wakes with breath holding, gasping, or choking. The bed partner or an observer reports habitual snoring or breathing interruptions in sleep. The patient has been diagnosed with hypertension, mood disorder, cognitive dysfunction, coronary artery disease, stroke, congestive heart failure, atrial fibrillation, or type 2 diabetes mellitus.
B. Polysomnography (PSG) or out‐of‐center sleep testing (OCST) demonstrates Five or more predominantly obstructive respiratory events (obstructive apnea, hypopnea, or respiratory effort‐related arousals) per hour of sleep during PSG or per hour of monitoring (OCST).
C. PSG or OCST demonstrates Fifteen or more predominantly obstructive respiratory events (apneas, hypopneas, or respiratory effort‐related arousals) per hour of sleep during PSG or per hour of monitoring (OCST).

## 4 MANAGEMENT OF SLEEP APNEA (CATHETER ABLATION OF AF AND OSA)

### 4.1 During catheter ablation procedure

Airway management to prevent obstructive apnea is essential to ensure the efficacy and safety of catheter ablation procedures. Catheter ablation with deep sedation has the potential risk of obstructive apnea due to glossoptosis. Obstructive apnea can lead to instability in the effects of sedation and analgesia, and may cause unexpected motion in patients, leading to inaccurate 3‐dimensional electroanatomic mapping. In addition, the shift in the position of the heart associated with dynamic diaphragmatic movements during and after an obstructive apnea episode might cause unstable catheter manipulation and the risk of cardiac tamponade.^45^ The substantial deep negative intrathoracic pressure during obstructive apnea may lead to air embolization through the blood access sheath.^46^ Although the effectiveness of catheter ablation under general anesthesia has been reported,^47^ general anesthesia is not widely performed in Japan because of the total time, cost, and insurance concerns associated with the procedure.^48^ The J‐CARAF study revealed that only 2.9% of the total 10,795 ablation procedures were performed under general anesthesia.^49^ Intraoperative monitoring and airway management are necessary for safe catheter ablation procedures. The Practice Guideline by the American Society of Anesthesiology recommends electrocardiography and blood pressure monitoring every 5 min by a medical staff member, other than the operator. Because it takes time for SpO_2_ to drop during apnea episodes under oxygen inhalation, end‐tidal carbon dioxide (EtCO_2_) monitoring with a capnometer is recommended for real‐time assessment of respiratory status during the procedure.^50^ Oral and nasal airway tubes and jaw elevation devices are useful for relieving upper airway obstruction.^16^, ^51^ CPAP is also useful in the prevention of obstructive apnea, and CPAP during catheter ablation restores the negative pressure of the LA by obstructive apnea.^52^

Post catheter ablation procedure.

Even after successful catheter ablation of AF, overlooked OSA might subclincally lead to the development of an arrhythmogenic substrate of AF in the atrium beyond the pulmonary vein and eventually cause recurrence as a challenging case of AF. Meta‐analysis of observational studies revealed that recurrence of AF after catheter ablation increased by 31% in patients with OSA in comparison to those without OSA^53^ and CPAP therapy after catheter ablation reduced AF recurrence by 42%^11^ in patients with OSA. Interestingly, the effect of CPAP on AF recurrence was confirmed regardless of the pulmonary vein isolation.^11^ It has been suggested that proarrhythmic mechanisms other than that related to pulmonary veins are involved in AF recurrence. Advanced structural remodeling in the atria beyond the PVs may lead to extra PV foci, which can initiate AF. Therefore, PV isolation is not sufficient in these patients, and additional ablation targeting extra‐PV foci might be needed to obtain better outcomes. Importantly, even after successful PV isolation, untreated OSA might cause the development of an arrhythmogenic substrate that eventually causes extra‐PV foci, leading to AF recurrence.

Recently, a randomized controlled trial showed that CPAP treatment did not reduce AF recurrence after catheter ablation in patients with paroxysmal AF. The AF recurrence‐free rate between the OSA‐CPAP and OSA standard care groups was 43%, as confirmed using an implantable loop recorder.^54^ It has been reported that sinus rhythm maintenance after pulmonary vein isolation was 65.7–87% according to data from a loop recorder implanted in patients with paroxysmal AF.^55^, ^56^ The patient population may include a more advanced arrhythmogenic substrate of AF, even in the paroxysmal type. The SLEEP‐AF study revealed that CPAP therapy can be reversible in terms of the electrophysiological properties associated with AF promotion.^57^ Therefore, CPAP therapy might be less effective in patients with more advanced structural remodeling.

## 5 FUTURE DIRECTION OF OSA SCREENING AND MANAGEMENT AS A COMPREHENSIVE TREATMENT OF AF

Cardiologists and arrhythmia specialists have many opportunities to care for patients with AF; however, there is a barrier to performing PSG in some hospitals, and systematic screening for OSA has not yet been established. In addition, some physicians as well as patients are less aware of the negative prognostic impact of OSA. Although the importance of comprehensive treatment of AF has been proposed, undiagnosed OSA is a modifiable risk factor, and appropriate therapeutic intervention is expected to enhance the effectiveness of AF treatments, including catheter ablation.^58^ Recently, various smartphone applications or mobile devices for self‐screening of OSA have been available and reported to be useful tool as a screening for moderate to severe OSA.^59^ Although physician should be cautions for making clinical diagnosis of OSA based on these devices, the novel technology will be able to facilitate screening of OSA. Moreover, establishing a comprehensive OSA care team for efficient diagnosis and treatment through close interdisciplinary collaboration between sleep‐disorder specialists and cardiologists is desired. OSA is one of the most expensive risk factors for both the screening and treatment of AF. Prospective clinical trials are needed to clarify the impact of OSA on the burden and outcome of AF, the benefit of OSA treatment, and the cost‐effectiveness of routine OSA screening.

## FUNDING INFORMATION

None.

## CONFLICT OF INTEREST

Author has no conflict of interest related to this review.
